# Evolution and mechanistic insights of platelet-derived products in temporomandibular joint regeneration

**DOI:** 10.3389/fcell.2026.1776592

**Published:** 2026-01-21

**Authors:** Cong Lin, Yutong Jin, Dan Li, Quanquan Yang, Tingyao Chen, Quan Chen, Mubin Zhang, Zhangbiao Long, Dongdong Fang

**Affiliations:** 1 Department of Oral and Maxillofacial Surgery, The Second Affiliated Hospital of Anhui Medical University, Hefei, China; 2 School of Pharmacy, Anhui University of Chinese Medicine, Hefei, China; 3 Department of Scientific Research, The Second Affiliated Hospital of Anhui Medical University, Hefei, China; 4 Department of Stomatology, Anhui Provincial General Prison Hospital, Hefei, China; 5 Department of Hematology, The First Affiliated Hospital of Anhui Medical University, Hefei, China

**Keywords:** fibrocartilage, immunomodulation, platelet-derived products, regenerative medicine, temporomandibular joint

## Abstract

Temporomandibular disorders (TMDs) and end-stage osteoarthritis are among the most common disabling diseases of the oral and maxillofacial region. Due to its unique fibrocartilaginous structure and limited vascularization, the temporomandibular joint (TMJ) possesses an extremely limited intrinsic regenerative capacity. Unlike conventional treatments that predominantly address symptoms, autologous platelet-derived products (APDs), such as platelet-rich plasma, platelet-rich fibrin, and concentrated growth factors, have been increasingly investigated for their biological roles in TMJ repair by mimicking natural healing mechanisms. This review summarizes the technical evolution of APDs and elucidates the molecular mechanisms promoting TMJ regeneration. Specifically, it discusses how APDs modulate the TMJ microenvironment by driving mesenchymal stem cell proliferation, directing chondrogenic differentiation, and resolving inflammation through immunomodulatory cascades. In addition, this review discusses the relevance of rheological properties for stage-specific clinical application and outlines translational considerations for the use of APDs in the management of TMDs.

## 1 Introduction

The temporomandibular joint (TMJ) is a complex, bilaterally coupled synovial joint with unique biomechanical demands (Gao et al., 2023). The progression of temporomandibular disorders (TMDs) is characterized by synovial inflammation, followed by progressive erosion of condylar cartilage and pathological remodeling of subchondral bone in advanced stages (de Oliveira et al., 2023). Current treatment strategies primarily focus on pain management and functional adaptation, but they have limited capacity to induce regeneration of damaged tissues. The condylar surface is covered by fibrocartilage, whose dense fibers resist shear stress and wear. However, this strength hinders its ability to repair damage. Fibrocartilage receives nutrients mainly from synovial fluid. Once injury or degeneration occurs, it lacks effective endogenous repair. Unlike long bones, the TMJ cannot self-repair through vascularization or callus formation (Detamore and Athanasiou, 2003; Cardoneanu et al., 2022). The core of modern biomanufacturing and tissue engineering is the automated integration of living cells, bioactive molecules, and biomaterial scaffolds (Groll et al., 2016). This synergy, known as the “three elements of regeneration,” enables the construction of functional tissues with hierarchical structures *in vitro* or *in vivo*. Autologous platelet-derived products (APDs) represent one such approach, as they combine growth factors, fibrin scaffolds, and cell-associated mediators within a single biological system. APDs are increasingly investigated as endogenous biologics in TMJ repair. They mimic physiological healing cascades, are abundantly available, and carry no risk of immune rejection.

**FIGURE 1:**
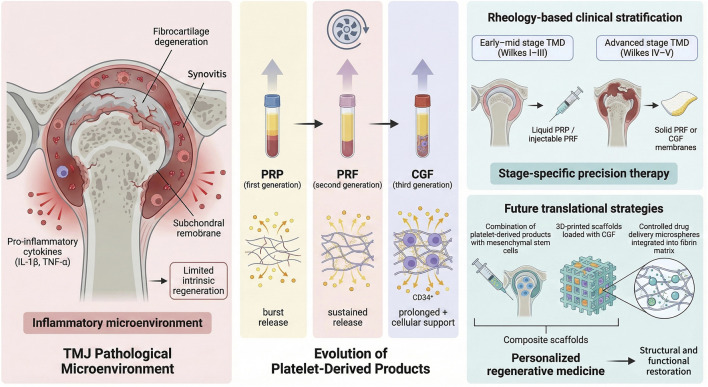
Evolution and rheology-guided stage-matching mechanisms of platelet-derived products for temporomandibular joint regeneration.

To clarify this strategy, Figure 1 presents the conceptual framework of this review. It integrates the pathological microenvironment of TMDs with the evolution of APDs. The framework further illustrates the rationale for rheology-guided, stage-specific clinical application, which is expanded upon in the following sections.

## 2 Process-dependent structural and functional differences

APDs, biologics rich in growth factors, have been widely applied in orthopedics, plastic surgery, and dentistry. The preparation of APDs represents a biologically instructive process rather than a mere technical protocol. Discrete variables, including centrifugal force, coagulation kinetics, and the integration of anticoagulants, fundamentally reshape the fibrin ultrastructure and dictate growth factor bioavailability. These parameters establish a distinct biophysical and biochemical milieu, governing how cells interpret mechanical cues and soluble signals within the joint microenvironment.

Differences among generations arise from variations in centrifugation mechanics and biochemical conditions during preparation. These products vary in fibrin structure, cellular composition, and growth factor release kinetics. The core of platelet-rich plasma (PRP) preparation is purification. PRP is produced through a two-step centrifugation process. Anticoagulants are added during preparation. Before use, exogenous activators are added to induce aggregation (Asiry et al., 2024). Adding activators causes PRP fibrin to form rigid tetrameric networks. This structure affects scaffold elasticity and stability. It also changes fibrin density, which directly impacts cell adhesion and migration within the matrix (Nagaraja et al., 2019). Platelet-rich fibrin (PRF) was developed later. It uses slow, physiological polymerization to form more elastic trimolecular junctions (Tsai et al., 2019; Mariani and Pulsatelli, 2020). This method avoids anticoagulants and allows sustained, gradual release of growth factors. It better mimics endogenous healing. PRF releases growth factors and cytokines gradually over at least 10 days. PRP gel shows different kinetics. After activation, it releases most growth factors and bioactive molecules immediately, with the majority released within 6–8 h (Liu R. et al., 2025).

Concentrated growth factors (CGF) build on PRF by introducing variable-speed centrifugation, achieving more precise separation (Salah et al., 2025). The physical separation creates a fibrin matrix denser and stronger than PRF. It also efficiently retains more CD34^+^ cells (Rodella et al., 2011). The presence of these stem cells gives CGF capabilities beyond a simple growth factor reservoir. It provides an endogenous cellular basis for inducing angiogenesis and tissue regeneration (Calabriso et al., 2021). The role of CD34^+^ cells in in situ chondrogenic and osteogenic differentiation is still debated. The CD34^+^ phenotype represents a phenotypic marker rather than a lineage-specific identifier, and most CD34^+^ cells containing products are heterogeneous. Available evidence supports a predominantly paracrine role in angiogenesis and immunomodulation. Compared with mesenchymal stem cells (MSCs), mechanistic and causal evidence supporting a direct role of CD34^+^ cells in TMJ regeneration remains limited and should therefore be interpreted with caution.

## 3 Role of platelet-derived products in TMJ repair

### 3.1 Regulation of the fibrocartilaginous phenotype

Fibrocartilage is characterized by the coexistence of type I and type II collagen in specific proportions (Zhang D. et al., 2023). Traditional cartilage repair strategies often view increased type II collagen as a standard indicator of regeneration (Song et al., 2022). Transforming growth factor-β (TGF-β) released by APDs can induce the expression of key chondrogenic transcription factors, such as Sox9. It also stimulates the production of type II collagen (Qin et al., 2023; Xiong et al., 2023; Chen et al., 2024). However, excessive stimulation by a single signal may induce chondrocyte hypertrophy, leading to pathological osteophyte formation (Epanomeritakis and Khan, 2024). APDs are rich in multiple growth factors, including TGF-β, insulin-like growth factor (IGF), platelet-derived growth factor (PDGF), and basic fibroblast growth factor (bFGF) (Ivanovska et al., 2022). By coordinating the actions of these developmental growth factor families, APDs help establish a finely tuned balance of fibrocartilaginous phenotype. Platelet-derived growth factor (PDGF) represents a crucial class of cytokines that plays a pivotal role in tissue repair and regeneration. The PDGF family comprises various isoforms. By binding to specific receptors (PDGFR-α and PDGFR-β), these isoforms activate downstream signaling pathways, such as PI3K/AKT, RAS/MAPK, and JAK/STAT (Obeagu, 2025). PDGF and bFGF stimulate fibroblast-like cells within the superficial zone to maintain type I collagen synthesis, ensuring the integrity of the articular surface (Zuo et al., 2023; Wu et al., 2024; Rolfes et al., 2025). PDGF and bFGF activate receptor tyrosine kinases on the surface of MSCs, thereby engaging downstream PI3K/Akt and RAS-MAPK/ERK signaling pathways (Giannotti et al., 2023). Activation of these pathways collectively enhances MSC survival, migration, and cell-cycle progression, leading to robust progenitor expansion and providing a sufficient cellular reservoir for subsequent tissue repair (Assi et al., 2024). PDGF promotes chemotaxis and mitotic activity of relevant tissue cells, including chondrocytes, fibroblasts, and MSCs. Furthermore, PDGF released from CGF effectively induces migration of endogenous synovial stem cells toward injury sites and enhances local matrix synthesis, playing a crucial role in the biological repair of articular disc perforations (Min et al., 2023; Shahbaz et al., 2024). TGF-β and IGF-1 work synergistically to promote proteoglycan synthesis and prevent matrix degradation by inhibiting the NF-κB pathway (Shi et al., 2019). In the TMJ osteoarthritis model, PRP-derived IGF-1 has been shown to attenuate chondrocyte endoplasmic reticulum stress and apoptosis, potentially through activation of the IGF-1R/PI3K/AKT signaling axis (Liu S. et al., 2025). In TMJ repair, PDGF promotes articular cartilage regeneration by regulating chondrocyte differentiation and matrix synthesis. For example, PDGF-AA upregulates the expression of Connexin 43 (Cx43) by activating the PI3K/Akt signaling pathway, thereby enhancing gap junction formation between osteoblasts, promoting intercellular communication and tissue regeneration (Zuo et al., 2023). Vascular endothelial growth factor (VEGF) promotes angiogenesis, indirectly supporting cartilage repair. In the early stages of injury, it supplies nutrients and oxygen to the new tissue (Yang et al., 2023).

The differentiation of chondrogenic MSCs by APDs is also regulated via the Wnt/β-catenin pathway. By upregulating endogenous, Wnt antagonists within the joint. APDs can attenuate β-catenin signaling induced by inflammatory mediators such as IL-1β (De Santis et al., 2018). This modulation helps prevent premature terminal differentiation of MSCs, maintaining the chondrogenic phenotype and reducing subchondral bone sclerosis and osteophyte formation.

Within the TMJ microenvironment, APD-derived TGF-βactivates Smad2/3 signaling, leading to upregulation of Sox9 in resident MSCs and thereby promoting commitment toward the chondrogenic lineage. In parallel, PDGF and bFGF stimulate the PI3K/Akt and MAPK/ERK pathways, respectively, driving MSC proliferation and cell-cycle progression to ensure adequate progenitor expansion prior to differentiation. IGF-1 further enhances MSC survival by suppressing apoptosis and sustaining cellular metabolic activity. Collectively, these coordinated signaling events establish a permissive regenerative niche that synchronizes stem-cell proliferation, lineage specification, and extracellular matrix synthesis, ultimately facilitating fibrocartilage regeneration within the TMJ. However, the TMJ environment is complex. Without structural support, cytokines alone are easily washed away by synovial fluid (Amini et al., 2022).

### 3.2 Remodeling the immune microenvironment

Macrophages, as key components of the immune system, show significant infiltration in the joint tissues of patients with TMJ osteoarthritis (TMJOA). Specific cytokines released by APDs can induce local macrophages to polarize from the pro-inflammatory M1 phenotype to the reparative M2 phenotype (Luo et al., 2021). Research has demonstrated that platelet-derived extracellular vesicles (PEVs) can transfer mitochondria to macrophages. This transfer drives a metabolic shift from glycolysis to oxidative phosphorylation. This metabolic reprogramming effectively promotes macrophage polarization toward the M2 phenotype while significantly suppressing M1 polarization (Wang et al., 2025). M2 macrophages secrete IL-10 and TGF-β, inhibiting the NF-κB pathway and reducing destructive factors such as IL-1β and TNF-α. This transforms the joint microenvironment from inflammation to regeneration (Anitua et al., 2024; Wen and Liu, 2025). CD34^+^ stem cells have also been shown to possess immunomodulatory functions, further enhancing this anti-inflammatory effect (Xiao et al., 2024). PRP-derived exosomes, enriched with various miRNAs and proteins, enhance synovial lymphatic function by activating the PI3K/Akt signaling pathway in lymphatic endothelial cells. This process facilitates the clearance of inflammatory cells and cytokines, which alleviates pain in a murine model of synovitis (Liao et al., 2025). Enriched in PRP-derived exosomes, miR-140-5p and miR-21 attenuate cartilage degradation and promote regeneration by targeting and suppressing the expression of matrix metalloproteinase-13 (MMP-13) and a disintegrin and metalloproteinase with thrombospondin motifs 5 (ADAMTS-5) (Jiang et al., 2024). Interleukins and tumor necrosis factor-α (TNF-α) in APDs can modulate immune cell activity through multiple pathways. IL-10, an anti-inflammatory cytokine, effectively inhibits macrophage polarization toward the M1 phenotype, thereby reducing the secretion of pro-inflammatory factors (Li X. et al., 2025). Results from mouse models show that PRP can reduce cartilage damage by inhibiting the dysregulation of matrix-related factors, including Sox9, Col2a1, Col10a1, and aggrecan. It also lowers the expression of inflammatory markers COX-2 and iNOS, and blocks phosphorylation of IκB and NF-κB in chondrocytes, mitigating doxorubicin-induced cartilage destruction (Zhao et al., 2021). Robust evidence supports the biological potency of APDs. However, clinical outcomes in the management of TMDs remain inconsistent across different studies. This discrepancy suggests that molecular mechanisms cannot fully explain therapeutic success. Instead, non-biological factors play a decisive role in clinical efficacy. Specifically, the rheological behavior and spatiotemporal stability of these materials within the joint cavity are critical to their performance.

## 4 Rheology-based clinical stratification and challenges in standardization

The TMJ represents a confined, fluid-rich, and mechanically active microenvironment. Within such a space, therapeutic efficacy depends not only on biological potency but also on material retention, spatial distribution, and resistance to synovial clearance. Consequently, the regenerative impact of platelet-derived products is intrinsically coupled to their rheological behavior. In this context, a “rheology-guided” therapeutic strategy refers to the rational selection of APDs based on their flow and deformation behavior within the TMJ. Rheological properties, including viscosity, flowability, and elasticity, critically influence injectability, spatial distribution, material retention, and mechanical stability (Russo et al., 2016). Viscosity primarily determines injection feasibility and post-injection dispersion, whereas flowability governs the ability of liquid formulations to penetrate synovial recesses and inflamed joint spaces. Elasticity and viscoelastic behavior reflect the capacity of fibrin-based matrices to maintain structural integrity, resist mechanical deformation, and function as provisional scaffolds under joint loading conditions (Fossati et al., 2024). Formulations with low viscosity and high flowability, such as PRP and injectable PRF (i-PRF), are better suited for minimally invasive intra-articular applications in early-stage TMDs dominated by synovitis.

### 4.1 Indication stratification for liquid and solid formulations

PRP, PRF, and CGF should not be regarded as simple substitutes with varying superiority. Instead, indication-specific stratification should be guided by their rheological properties. In early-to mid-stage TMDs with preserved joint structure but active synovitis (Wilkes stages I–III), liquid PRP and i-PRF, owing to their superior flowability, are suitable options for joint lavage and intra-articular injection (Fossati et al., 2024). These formulations can rapidly penetrate synovial folds, exerting anti-inflammatory and lubricating effects. As a result, they relieve pain and improve mouth opening (Al-Moraissi et al., 2019; Zotti et al., 2019). In contrast, in advanced cases involving disc perforation or severe osteochondral destruction (Wilkes stages IV–V), liquid formulations cannot provide sufficient structural support (Liu K. et al., 2024). Solid PRF and CGF membranes, owing to their denser fibrin networks and adequate suture strength, serve as potential surgical patch materials (Mozzati et al., 2022). These solid scaffolds not only physically seal perforations but also act as regenerative matrices that promote thickening and remodeling of the fibrocartilaginous layer. CGF membranes are superior to PRF in generating a thicker and better-organized fibrocartilaginous layer (Lee et al., 2020). PRP attenuates joint capsule fibrosis by inhibiting the TGF-β1/Smad2/3 pathway and reducing fibrosis markers in synovial fibroblasts (Zhang Y. et al., 2023). Dense fibrin scaffolds protect growth factors from rapid degradation through the combined effects of physical barrier formation and electrostatic adsorption, thereby maintaining effective therapeutic concentrations over an extended period (Li Y. et al., 2025). This mechanistic difference is reflected in clinical outcomes. A network meta-analysis showed that PRP was superior to PRF and hyaluronic acid in improving mouth opening at 1 month postoperatively. However, PRF demonstrated more significant and sustained improvements at 3 and 6 months (Xu et al., 2023). This temporal variation in efficacy highlights the importance of tailoring rheological properties to the TMD stage instead of applying a uniform injection approach.

### 4.2 Translational bottlenecks and standardization challenges

Although clinical applications of APDs are expanding, their use in the treatment of TMDs remains limited due to methodological heterogeneity and poor biostandardization. These factors lead to highly inconsistent outcomes. Several registered trials identified in a search of ClinicalTrials.gov (December 7, 2025) evaluate APDs for TMDs management. Their study designs and primary outcomes are summarized in Table 1. Current clinical studies often lack standardized reporting criteria for preparation parameters. There are notable variations in parameters such as centrifuge speed, rotor radius (Tovar et al., 2021), rest time (Wei et al., 2022), and collection tube material (Wei et al., 2024) across different studies. Very few studies have quantified the concentrations of growth factors, platelet recovery rates, and leukocyte content in the final product (Gomri et al., 2022). The biological activity of PRP correlates closely with platelet enrichment, yet the optimal platelet concentration remains undefined in clinical practice. Leukocyte-rich (LR-PRP) and leukocyte-poor (LP-PRP) formulations exhibit distinct mechanisms of action, where LR-PRP is preferentially indicated for tissues with high immunomodulatory demands, whereas LP-PRP favors scarless healing (Rath et al., 2025). A Swiss-developed automated closed separation system uses thixotropic separation gel technology to consistently prepare leukocyte-poor PRP (LP-PRP) with a platelet recovery rate of ≥90%. Clinical studies demonstrate that in the treatment of osteoarthritis, the coefficient of variation in patient pain score improvement is reduced to within 15%. The introduction of red blood cells during the preparation process can lead to their lysis within the joint cavity, releasing iron ions. This subsequently induces oxidative stress and exacerbates cartilage damage (Leuci and Dargaud, 2023). To prevent residual erythrocytes from inducing joint inflammation, preparation protocols must restrict their content to within 0.5% through the optimization of centrifugation parameters or the implementation of leukocyte filtration techniques (Rieske et al., 2020). Whether non-standardized APD preparations accelerate the ferroptosis process in advanced TMJ osteoarthritis remains a critical question, requiring rigorous mechanistic investigation and clinical validation. Exogenous activators in PRP can trigger immune responses. These reactions vary among individuals (Niemczyk et al., 2024). These immune responses are frequently linked to the high concentrations of growth factors and inflammatory mediators in APDs. Future translational research should follow the Minimum Information for Studies Evaluating Biologics in Orthopedics (MIBO) guidelines. A quality control system based on final bioactive dosage is needed. This system should not rely solely on preparation methods (Laohajaroensombat et al., 2023). The MIBO guidelines were developed to improve transparency and reproducibility in studies of biologic therapies, including platelet-derived products. However, inconsistent adherence in APDs research still hampers cross-study comparability and dose-response interpretation, underscoring the need for biologically prioritized quality control beyond reporting standards. At present, seven different biological classification systems have been proposed to simplify the use of APDs. These systems have not been widely adopted in clinical practice. Such systematic biological and quantitative analyses are time-consuming and costly. They rely on specialized equipment and laboratory support, making them impractical for most clinicians to implement at an individual level (Popescu et al., 2021). Patient-related factors, such as age, systemic diseases, and medication use, also significantly affect the quality and activity of APDs (Noguchi et al., 2024). This complexity suggests that the future clinical application of APDs may ultimately require the establishment of personalized quality standards.

**TABLE 1:** Summary of registered clinical trials investigating platelet-derived products for the treatment of temporomandibular disorders.

APDs	Intervention(s)	Intervention details	Enrollment	Inclusion criteria	Primary outcome	Status	ClinicalTrials.gov identifier
PRP	PRP	160 rpm; 5 min; mixing with iFuge D06	Not reported	ICOP 2020 diagnosis of TMJ pain	Mandibular: Abduction protrusion, lateral movement	Completed	NCT06530745
	PRP + HA	800rpm; 8 min; mixing with HA and calcium Chloride-rich activator	Not reported	MRI and CT	TMJ arthralgia (VAS)	Active, not recruiting	NCT06457698
	PRP	Not reported	120	DC/TMD protocol	TMJ arthralgia (VAS)	Completed	NCT03371888
	PRP	3000 rpm 10 min	Not reported	Not reported	TMJ arthralgia (VAS); MMO	Recruiting	NCT06839326
	PRP	Not reported	20	Wilkes stages IV and V	MMO; TMJ arthralgia (VAS)	Completed	NCT04936945
PRF	iPRF	Not reported	Not reported	Not reported	TMJ arthralgia (VAS); Mandibular abduction	Completed	NCT05883982
	iPRF	Not reported	Not reported	MRI and CT	TMJ arthralgia (VAS)	Completed	NCT07188974
	iPRF	Not reported	Not reported	TMJOA,MMO≤35 mm	TMJ arthralgia (VAS)	Completed	NCT04810923
	iPRF	Not reported	Not reported	Wilkes stages III、IV and V	TMJ arthralgia (VAS)	Completed	NCT06766851
CGF	Liquid phase CGF	Not reported	24	MMO <40 mm,MR	MMO Disc-condyle relationship Myeloperoxidase enzyme activity	Completed	NCT04557878

Data were retrieved from the ClinicalTrials.gov database on December 7, 2025. Abbreviations: TMD, temporomandibular disorder; PRP, platelet-rich plasma; PRF, platelet-rich fibrin; HA, hyaluronic acid; MRI, magnetic resonance imaging; CT, computed tomography; VAS, visual analog scale; MMO, maximum mouth opening; TMJOA, temporomandibular joint osteoarthritis; DC/TMD, Diagnostic Criteria for TMD; ICOP, international classification of orofacial pain; NCT, national clinical trial identifier.

## 5 Summary and outlook

Minimally invasive treatment is central to the future of therapy for TMDs. Injectable PRF combines the convenience of liquid injection with the support of a solid scaffold (Sherif et al., 2025). A network meta-analysis indicates that i-PRF, as an intra-articular injection, can effectively relieve TMD symptoms and restore joint function. Its therapeutic effects typically become evident within 3 months post-procedure (Zhang et al., 2024). Future research should focus on optimizing its coagulation time and rheological properties to better adapt to the confined space of the temporomandibular joint cavity.

Injectable systems are effective for early- and mid-stage TMDs, but individual APDs show mechanical limitations, especially when repairing large, complex tissue defects. Combining APDs with other therapies, such as exogenous MSCs, is an emerging trend that produces a synergistic osteoinductive effect (Hu et al., 2021; Palermo et al., 2022; Everts et al., 2025). In animal models, transplanting PRP embedded with MSCs into a large area of TMJ condylar cartilage defect can induce the formation of cartilage-like tissue and repair the joint surface. This is simultaneously accompanied by subchondral bone filling without the occurrence of bone sclerosis (Gomez et al., 2020).

Three-dimensional printing can construct polycaprolactone or collagen scaffolds filled with CGF as a bioactive ink, providing sustained mechanical support to compensate for fibrin’s rapid degradation. It also enables the spatiotemporally controlled release of growth factors (Hao et al., 2022; Liu X. et al., 2024). Small-molecule anti-inflammatory drugs can be encapsulated in microspheres. These microspheres are then integrated into APDs. This delivery system enables dual-phase sequential regulation. It controls both the anti-inflammatory and regenerative processes (Liu L. et al., 2024).

APDs are valuable tools for TMD therapy. Beyond better preparation, current advances focus on modulating the regenerative microenvironment. Realizing clinical efficacy requires a deeper understanding of TMJ fibrocartilage biology. Standardized protocols and rheology-based criteria for indications are also essential. Future studies should develop functionalized composite scaffolds to address the mechanical and biological demands of the TMJ. Such systems aim to restore both joint structure and function.
